# Monitoring Variables Influence on Random Forest Models to Forecast Injuries in Short-Track Speed Skating

**DOI:** 10.3389/fspor.2022.896828

**Published:** 2022-07-14

**Authors:** Jérémy Briand, Simon Deguire, Sylvain Gaudet, François Bieuzen

**Affiliations:** Institut National du Sport du Québec, Montréal, QC, Canada

**Keywords:** data mining, machine learning, high performance, sport injury prevention, modeling

## Abstract

Injuries limit the athletes' ability to participate fully in their training and competitive process. They are detrimental to performance, affecting the athletes psychologically while limiting physiological adaptations and long-term development. This study aims to present a framework for developing random forest classifier models, forecasting injuries in the upcoming 1 to 7 days, to assist the performance support staff in reducing injuries and maximizing performance within the Canadian National Female Short-Track Speed Skating Program. Forty different variables monitored daily over two seasons (2018–2019 and 2019–2020) were used to develop two sets of forecasting models. One includes only training load variables (TL), and a second (ALL) combines a wide array of monitored variables (neuromuscular function, heart rate variability, training load, psychological wellbeing, past injury type, and location). The sensitivity (ALL: 0.35 ± 0.19, TL: 0.23 ± 0.03), specificity (ALL: 0.81 ± 0.05, TL: 0.74 ± 0.03) and Matthews Correlation Coefficients (MCC) (ALL: 0.13 ± 0.05, TL: −0.02 ± 0.02) were computed. Paired *T*-test on the MCC revealed statistically significant (*p* < 0.01) and large positive effects (Cohen d > 1) for the ALL forecasting models' MCC over every forecasting window (1 to 7 days). These models were highly determined by the athletes' training completion, lower limb and trunk/lumbar injury history, as well as sFatigue, a training load marker. The TL forecasting models' MCC suggests they do not bring any added value to forecast injuries. Combining a wide array of monitored variables and quantifying the injury etiology conceptual components significantly improve the injury forecasting performance of random forest models. The ALL forecasting models' performances are promising, especially on one time windows of one or two days, with sensitivities and specificities being respectively above 0.5 and 0.7. They could add value to the decision-making process for the support staff in order to assist the Canadian National Female Team Short-Track Speed Skating program in reducing the number of incomplete training days, which could potentially increase performance. On longer forecasting time windows, ALL forecasting models' sensitivity and MCC decrease gradually. Further work is needed to determine if such models could be useful for forecasting injuries over three days or longer.

## Introduction

Sports performance is complex and influenced by many factors such as genetic heritage, training level, trainability (Issurin et al., 2005), sportive technique, nutrition, psychological state, and health status (Gould et al., 1999; D'Isanto, 2019). Injuries, which we define as any physical or mental complaint limiting the athletes' ability to participate fully in training and competition (Meeuwisse et al., 2007; Clarsen et al., 2013), are part of the factors affecting sports performance (Renström and Johnson, 1985; Clarsen et al., 2013, 2021; D'Isanto, 2019). Within high-performance systems, injuries are common (Lian et al., 2005; Waldén et al., 2005; Clarsen et al., 2010), potentially because of the high demands of competitive sports, requiring the athletes to push their body to its limit (Soligard et al., 2016). They restrict the athletes' ability to progress, affecting performance, long-term development, and athlete retention while adding financial costs (Bahr and Krosshaug, 2005; Bahr et al., 2018).

Injuries are complex and influenced by many interconnected factors (Meeuwisse et al., 2007; Halson, 2014; Bittencourt et al., 2016). We can regroup these factors into three main categories. The first one refers to the stress applied to the athlete. Previous studies extensively explored the impact of external and internal training load (stress imposed on and perceived by the athlete) (Foster et al., 2017) on injuries, often using acute to chronic work ratio (ACWR) (Hulin et al., 2014; Stares et al., 2017). The second category refers to the athletes' predispositions that will affect the stress response, such as their physical and psychological health status, age, injury history, flexibility level, fatigue, muscle strength, and imbalances (Meeuwisse et al., 2007; López-Valenciano et al., 2018). The last category is the athletes' stress response. According to Selye's general adaptation syndrome (Selye, 1950), the stress response can lead to positive adaptations (Eustress), such as sports performance, or negative adaptations (distress), such as injuries. This stress response (eustress or distress) can, in turn, modify the athletes' predisposition and influence their future response to stress exposure. We hypothesize that proper monitoring of various quantitative variables from each of the three categories of factors influencing injuries and sports performance, combined with efficient association and observational techniques, could help predict the athletes' future injuries.

Many interrelated variables influence injuries, which complexifies their association with risk factors (Meeuwisse et al., 2007; Bittencourt et al., 2016; Van Eetvelde et al., 2021). To tackle this problem, studies have started using machine learning algorithms (Van Eetvelde et al., 2021). Machine learning is a tool that uses computers to “learn” complex relationships from empirical data and that establishes a mathematical link between a large number of covariates and a target variable of interest (Cabitza et al., 2018). Among the studies stated in Van Eetvelde's (Van Eetvelde et al., 2021) systematic review of machine learning applications to sports injuries, different types of variables were monitored: (1) sports background (McCullagh and Whitfort, 2013; López-Valenciano et al., 2018; Ayala et al., 2019); (2) psychological measurements (McCullagh and Whitfort, 2013; López-Valenciano et al., 2018; Ayala et al., 2019); (3) neuromuscular measurements (López-Valenciano et al., 2018; Ayala et al., 2019); (4) workloads (McCullagh and Whitfort, 2013; Whiteside et al., 2016; Rossi et al., 2017; Thornton et al., 2017; Carey et al., 2018); (5) previous injury background and health status (McCullagh and Whitfort, 2013; Rossi et al., 2017; López-Valenciano et al., 2018; Ruddy et al., 2018); (6) genetic markers (Rodas et al., 2019); and (7) personal data (age, body mass, anthropometric measurements, demographic) (McCullagh and Whitfort, 2013; Whiteside et al., 2016; Ruddy et al., 2018; Oliver et al., 2020; Rommers et al., 2020). Each type of variable helps quantify one of the three categories of factors influencing injuries. Thus far, only the McCullagh and Whitfort (2013) study has regrouped predictors from the three categories of factors influencing injuries and sports performance.

Short-track speed skating is a highly interactive middle distance sport, which consists in performing multiple laps on the ice at high speed, including tight corners and up to nine athletes in a single race (Hesford et al., 2012; Menting et al., 2019; Konieczny et al., 2020). It has been studied mostly for its biomechanical aspect (Hesford et al., 2012; Kim et al., 2019; Konieczny et al., 2020), as well as its strategic and pacing dimensions (Haug et al., 2015; Konings et al., 2016; Konings and Hettinga, 2018; Menting et al., 2019). This sport induces important leg asymmetries (Hesford et al., 2012; Konieczny et al., 2020), which are suspected to put the athletes particularly at risk of sustaining added injuries in the lower limb regions (Konieczny et al., 2020). Within the Canadian National Female Short-Track Speed Skating Program, over the 2018-2019 and 2019-2020 seasons, athletes have lost on average 75 ± 45 training or competition days due to injuries, which represents 12 ± 7% of lost training and/or competition opportunities per athlete, over two seasons. On average, 2 days were missed per injury. Implementing a framework helping the performance support staff detect and prevent injuries and their risk factors is required to maximize performance within the Canadian National Female Short-Track Speed Skating Program.

Using a random forest algorithm, shown to be efficient on multiple features classification problems, such as injury prediction (Breiman, 2001), the current study aims to: (1) provide a narrative on a data mining framework (Adriaans and Zantinge, 1997) within the Canadian National Female Short-Track Speed Skating Program; (2) demonstrate that random forest classifiers forecasting injuries could benefit the performance support staff; and (3) highlight the variables of importance in the injury forecasting process. Finally, the study aims to (4) demonstrate how the combination of monitored variables from each of the three categories of factors influencing injuries improves the ability to forecast potential injuries compared to models only built using training load variables.

## Materials and Methods

### Forecasting

Van Eetvelde et al.'s review (Van Eetvelde et al., 2021) presents mostly prospective case-control studies (McCullagh and Whitfort, 2013; Rossi et al., 2017; Thornton et al., 2017; Carey et al., 2018; López-Valenciano et al., 2018; Ruddy et al., 2018; Ayala et al., 2019; Oliver et al., 2020; Rommers et al., 2020), analyzing data retrospectively and predicting injuries based on the athletes' features value on a given day. In this prospective case-control study, we predicted the athletes' injury status in the upcoming 1 to 7 days. This will help the performance support staff anticipate athletes' upcoming injuries over the next training micro-cycle, which corresponds to 7 days within the Canadian National Female Short-Track Speed Skating Program.

### Data Mining Approach

**Figure 1:**
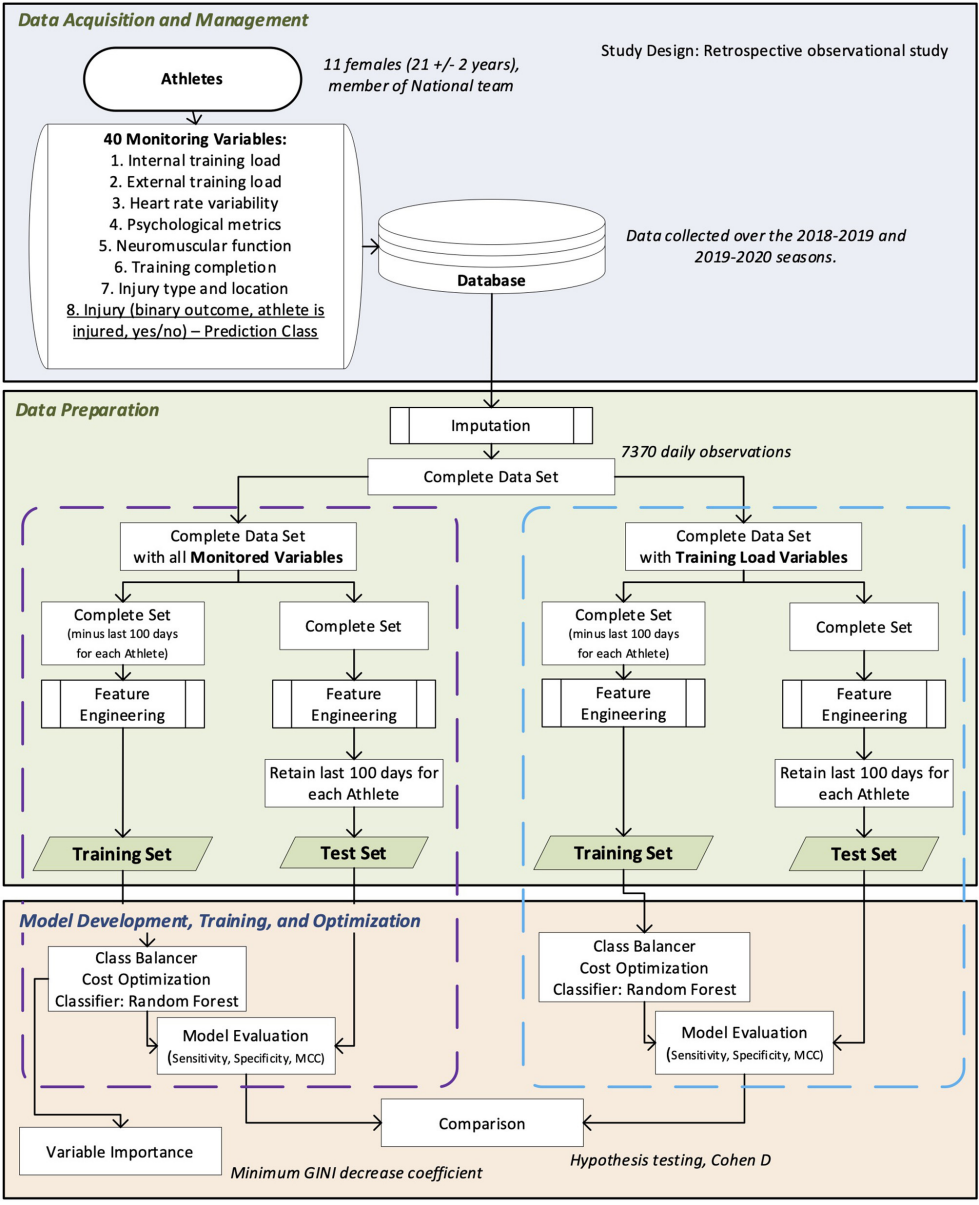
Overview of the data mining framework to develop and evaluate random forest forecasting models.

We put a data mining system (Adriaans and Zantinge, 1997) in place within the Canadian National Female Short-Track Speed Skating Program to train and evaluate the random forest injury prediction models. Figure 1 provides a schematic of the different steps of data acquisition, preprocessing, feature engineering, and model training and evaluation. Each step presented in Figure 1 will be explained in the following subsections.

### Athletes

We collected data over the 2018–2019 and 2019–2020 seasons. Eleven women, members of the Canadian Short-Track Speed Skating National Team, consented to take part in the study. Every athlete signed an “athlete consent form” allowing the use of their data for research purpose and program evaluation. The consent form was approved according to provincial legislations and ethical recommendations. Sensitive information that can directly identify the athletes (e.g. names, addresses) were safeguarded and maintained under controlled conditions according to provincial laws. The athletes went through a familiarization and education process to ensure data were reported in a standard and appropriate way. Table 1 describes the characteristics of the athletes who participated in the study.

**Table 1:** Athletes characteristics summary.

**Athletes characteristics**	**Mean ±standard** **deviation**	**Range**
Age (years)	21 ± 2	18–24
World ranking	27 ± 19	2—Not ranked
Experience on the national team (years)	4 ± 2	1–9

### Conceptual Model of Injuries and Monitored Data

**Figure 2:**
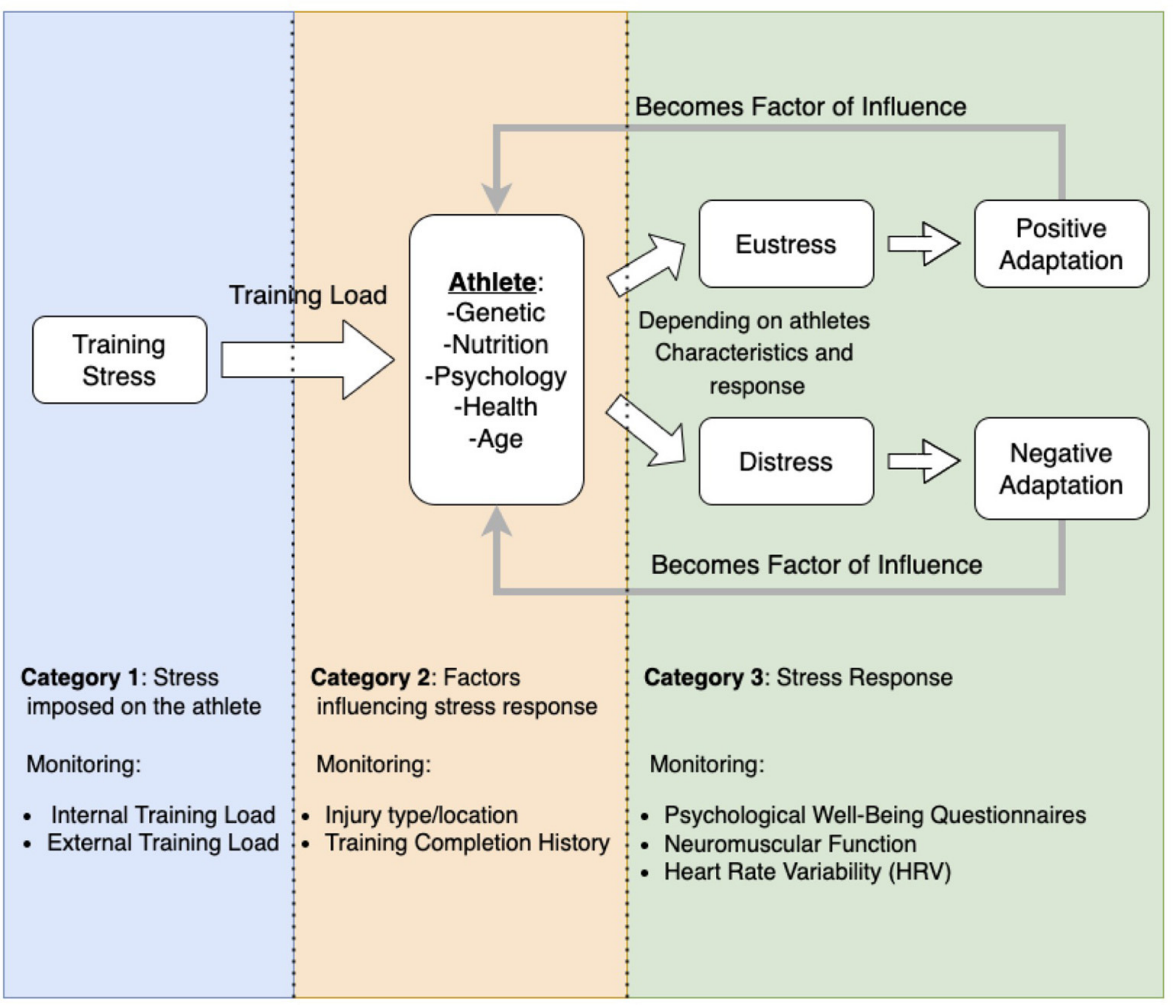
Conceptual framework of the factors influencing sport performance and injuries and their inter-relation.

The conceptual model of injuries and sports performance, presented in Figure 2, was created to guide our interpretation of injuries and their underlying mechanisms and provide adequate perspective on the influence of the three categories of factors on injuries. It is inspired by Selye's general adaptation syndrome (Selye, 1950), Meeuwisse et al. (2007) dynamic model of sports injury etiology, and D'Isanto (2019) factors influencing sports performance. The model (Figure 2) guided the choice of variables to monitor. The amount of stress imposed on the athlete was quantified by monitoring the external (training stress imposed on the athlete) and internal training load (training stress perceived by the athlete) (Foster et al., 2017). Factors influencing stress response were monitored through psychological wellness questionnaire scores (Junge, 2000; Shrier and Hallé, 2011), tracking of athletes' injury type and location history, and monitoring of their history of training completion (ability to complete the prescribed training). The stress response was quantified through the tracking of the athletes' heart rate variability (HRV) (Goessl et al., 2017) and neuromuscular function (Gathercole et al., 2015).

We tracked 40 different variables through the monitoring process, grouped into eight subsets, which are described in Table 2. Combining the data from the 11 athletes over the two seasons provided a total cumulative dataset of 7,370 daily observations (a daily observation regroups the 40 monitored variable values with the associated injury outcome). The monitored data were stored in a database.

**Table 2:** Description of the different variables, their measurement frequency, and the strategies used to replace missing values.

**Variable**	**Description and measurements**	**Measurement frequency**	**Missing values replacement strategy**
External training load	Number of laps on the ice rink performed for each training by the athletes in different intensity zones.	Every training on ice	Main cause: Athletes did not train. Missing values replaced with a zero.
Internal training load	Athletes qualitatively assigned fatigue perception on a scale of 0 to 10, which was multiplied by the session duration, in minutes, to provide an internal load score referred to as sFatigue (Dunbar et al., 1992)	After every training session	Same as external training load.
Psychological wellbeing metrics	Athletes provided an assessment, on a scale of 0 to 100, of their levels of stress, energy, happiness, mood, motivation, performance stress, and sleep quality over the three previous days (Junge, 2000; Shrier and Hallé, 2011).	3 times a week	Main Cause: non-daily measurement frequencies. Replaced with the most recent measurement.
Heart rate variability	Resting heart rate variability (HRV) taken using a heart rate monitoring belt (Polar H10, Finland) connected HRV4Training (Altini et al., 2017). Variables measured: resting heart rate, high frequency power (hf), low frequency power (lf), pnn50 (Seyd et al., 2008), RMSSD (Wang and Huang, 2012), sdnn (Wang and Huang, 2012; Goessl et al., 2017).	Every 3 days	Same as psychological wellbeing metrics.
Neuromuscular function	Counter movement jumps (CMJ) performed on force plates. Variables measured: contraction time, flight time to contraction time ratio, jump height (from flight time and impulsion), takeoff velocity, flight time to contraction time ratio, height (from impulsion), height (from flight time) and flight velocity (Gathercole et al., 2015).	3 times a week	Same as psychological wellbeing metrics.
Injury type and location	Each time athletes reported injuries they specified injury body location (head/neck, trunk, trunk/lumbar, lower limb, upper limb) and the type (bone, muscle and tendon, joint and ligament, skin, brain/spinal cord/peripheral nervous system, other).	Each time an injury was reported	Main cause: Athlete was not injured. Replaced with a zero.
Training completion	The athletes ranked the level of training completion according to four factors: 0: training completed without injury/illness, 1: training completed with injury/illness, 2: training could not be completed because of injury/illness and 3: The athlete could not train at all because of injury/illness.	After every training session	Main cause: Athlete did not train. Missing values replaced with a zero. In the cases where athletes could not train because of injury or illness, the corresponding health status was validated by the medical team.
Injury (Target variable: Forecast 1 to 7 days)	The injury status, by definition (Meeuwisse et al., 2007; Clarsen et al., 2013) refers to the *Training completion* variable value in a future time window of 1 to 7 days. A *Training completion* of 0 was labeled as a “non-injured” athlete, while a *Training completion* of 1, 2 and 3 was labeled as an “injured” athlete. Models were trained to predict this variable.	Every day	Variable derived from the *Training completion* When the Injury variable is defined there are no missing values left.

*For the missing values and replacement strategies, the main cause of missing values is reported in the third column of the table. A close follow-up was made with the performance support staff to verify all information for specific situations, such as athletes omitting to report due to injuries*.

### Data Preprocessing: Missing Values Imputation and Feature Engineering

The monitoring process implies inevitable missing values (Benson et al., 2021). We observed three causes for missing values in the dataset: (1) the athletes did not train on a given day; (2) some variables were not measured daily; and (3) the athletes omitted to report data. Even if all athletes who participated in the study had participation rates ranging from 77 to 100% and averaging 90%, some daily instances were incomplete and displayed, for some variables, missing values. Imputation strategies had to be employed and are reported, for each monitored variable subset, in Table 2. Replacing missing values by the most recent measurement was the preferred imputation strategy for the psychological wellbeing metrics, neuromuscular function, and HRV variables, which were measured every 2 to 3 days. Although not perfect, the imputation strategy is easy to implement, avoids information leakage (Van Eetvelde et al., 2021), and provides indicators of the stress response variables over a short time frame. It is the most simplistic single imputation technique described by Benson et al. (2021).

Injuries were forecasted through a feature-based approach. New variables were computed from the athlete's monitored variables history, stored in the database, to reflect injuries' time dependence (Clarsen et al., 2013; Bittencourt et al., 2016) and improve the models' forecasting performances. We calculated the exponentially weighted moving average (EWMA) over the last 7 and 28 days (referred to as the acute and chronic indices) for each monitored variable. These time frame periods were chosen based on previous studies exploring the association between these indices and injuries (Gabbett and Jenkins, 2011; Hulin et al., 2014; Gabbett et al., 2016). It also corresponds to the microcycles (7 days) and mesocycles (28 days) used for the athletes' global periodization. The acute to chronic work ratio (ACR) (Gabbett and Jenkins, 2011; Hulin et al., 2014; Gabbett et al., 2016) is an additional feature engineered for all the variables. The EWMA ratios are a standard forecasting practice to improve the forecasting models' performances (Holt, 2004). In addition, we computed the acute to chronic difference (ACD), inspired by Banister's training impulse concept (Banister et al., 1975; Jovanovic, 2018).

For each variable, to quantify the influence of the past mesocycles and microcycles, we computed rolling means, standard deviations, and maximums over time windows of 7, 14, 21, and 28 days. At the end of the feature engineering process, a single daily observation is constituted of 1014 features associated with the athletes' injury status (injured or not injured).

### Training and Test Sets

To make predictions, the random forest algorithms were “trained” on available data and “learned” a set of rules that allow appropriate classification (injured or not injured), given the set of features provided as input. This set of features was composed of: (1) monitored variables on any given day; (2) imputed variables (if some monitored variables were missing); (3) engineered features based on the athletes' monitored variables history stored in the database.

To evaluate the classifiers' ability to replicate their predicting performances, they were evaluated on a test set, *i.e*., a set of unseen data (not used in the models' training process). Two sets of forecasting models were developed. One including only training load variables (TL), and a second (ALL) combining a wide array of monitored variables (neuromuscular function, heart rate variability, training load, psychological wellbeing, past injury type, and location). From the 7,370 available daily instances, we created two test sets of 1,100 instances, composed of each of the 11 athletes' last (chronologically) 100 daily instances. The ALL-Test-Set was composed of each of the 40 monitored variables and their respective engineered features, based on each athlete's complete data history, for a total of 1,014 features, while the TL-Test-Set was composed only of internal and external training load monitored variables and their corresponding engineered features, for a total of 182 features. With the remaining 6,270 daily instances, we created two training sets: the ALL-Training-Set composed of the same features as the ALL-Test-Set and the TL-Training set composed of the same features as the TL-Test-Set. As depicted in Figure 1, the test sets feature engineering was performed on the athletes' complete data history. The training sets feature engineering was only performed on the remaining 6,270 daily observations. This process replicates the way data will be handled to make predictions once the models are implemented in the training environment and avoid data leakage, where information of the test set would leak in the training set, leading to overfitting and biased interpretation of the models' evaluation (Van Eetvelde et al., 2021). Two distinct data sets (ALL and TL) were created to assess the impact of building forecasting models composed of variables from each of the three components of the injury conceptual model (ALL) in comparison to models composed of variables only reflecting the stress applied to the athlete (TL).

### Models Training

Two sets of models were trained using the *Waikaito Environment for Knowledge Analysis (WEKA)* (Hall et al., 2009). WEKA provides built-in functions to facilitate classifications of imbalanced datasets, such as the one in this study, where non-injured outweighs injured daily observations. To improve the random forest injury classifier, the injured and non-injured instances of the test set were balanced using WEKA's *class balancer* filter, which applies a weight function to the instances such that injured and non-injured instances display a similar total sum of weight (Hall et al., 2009). A cost function was also introduced using WEKA's *cost-sensitive classifier* function. The function penalizes injury misclassifications through the random forests training process, which improves injury forecasting performances (Hall et al., 2009). Default setups were used for all of WEKA's built-in functions. The ALL forecasting models were trained on the ALL Training Set, and the TL forecasting models were trained on the TL Training Set. For each set, seven classifiers were trained to predict whether the athletes would be injured in an upcoming time frame of 1 to 7 days, respectively. The random forest algorithm introduces randomness, notably through the bootstrap aggregating process (Breiman, 2001). To account for the classifiers' random factors, the training of each classifier was performed 30 times for each of the seven forecasting time frames. At the end of the training process, we obtained, for both sets of models (ALL and TL), 30 trained random forest classifiers for each forecasting time window (1 to 7 days).

### Models Evaluation

Each instance of the test-set was provided as input to the forecasting models, and the trained classifier generated predictions on the upcoming injury status (on a time frame of 1 to 7 days depending on the classifier). To obtain a confusion matrix, each prediction was compared to the true outcome, which is already known. Confusion matrices compile True Positives (TP), False Positives (FP), False Negatives (FN), and True Negatives (TN) (Visa et al., 2011). We computed the following three metrics from confusion matrices that summarized each of the 30 models' predictive performances for every forecasting time window (1 to 7 days).

The sensitivity.


$$\begin{array}{l} Sensitivity=\frac{TP}{TP+FN} \end{array}$$

The specificity


$$\begin{array}{l} Specificity=\frac{TN}{TN+FP} \end{array}$$

the Matthews Correlation Coefficient (MCC) (Matthews, 1975).


$$MCC=\frac{TP\cdot TN-FP\cdot FN}{\sqrt{\left(TP+FP)(TP+FN)(TN+FP)(TN+FN\right)}}$$

The sensitivity and specificity are commonly used evaluation metrics and were computed to compare our models with existing sports injury prediction models (Van Eetvelde et al., 2021). The MCC (Matthews, 1975) is considered one of the best methods to evaluate binary classifiers (Powers, 2020). It is a wellbalanced metric that can be used even if the two classes are unbalanced (Boughorbel et al., 2017). An MCC of 1 indicates complete agreement between the predictions and the observation, and inversely, an MCC of −1 denotes complete disagreement (Boughorbel et al., 2017). An MCC of 0 would be considered a model where the predictions were made randomly (Boughorbel et al., 2017). The unbalanced nature of the data set is the reason why accuracy (Yin et al., 2019) was not used as an evaluation metric. In our case, out of the total 7,370 daily observations, only 12% constitute injuries. Therefore, even if the models were always to predict non-injured athletes, the accuracy would remain relatively high (~0.88) and would not provide valuable information to the practitioners. We computed the mean and standard deviation of the 30 sensitivity scores, 30 specificity scores, and 30 MCC of every forecasting time window. At the end of the evaluation process, we obtained three evaluation metrics (mean ± standard deviation) for each model and each forecast window (1 to 7 days) for both the ALL and TL forecasting models.

### Models Comparison

To analyse the effect of the forecasting model types (ALL or TL) and forecasting time windows (1 to 7 days) on each evaluation metric (sensitivity, specificity, MCC), two-way ANOVAs were performed on each metric. In addition to ANOVAs, paired *T*-tests (Meek et al., 1987) were performed on each separate forecasting window to evaluate the effect of the model type on the evaluation metrics, with the null hypothesis that both forecasting models (ALL and TL) evaluation metrics were equal at a significance level of *p* < 0.01. In addition, we computed Cohen effect sizes (Cohen, 1994; Fritz et al., 2012) to quantify the difference between the two types of model evaluation metrics.

### Variables of Importance

Random forest classifiers enable highlighting variables of importance, which have a practical application for the performance support staff, trying to understand better the causes and mechanisms of injuries (Meeuwisse et al., 2007; Bittencourt et al., 2016; Van Eetvelde et al., 2021). The higher the random forests' minimum GINI decrease coefficient, the greater the importance of the variable in the model (Archer and Kimes, 2008).

## Results

**Figure 3:**
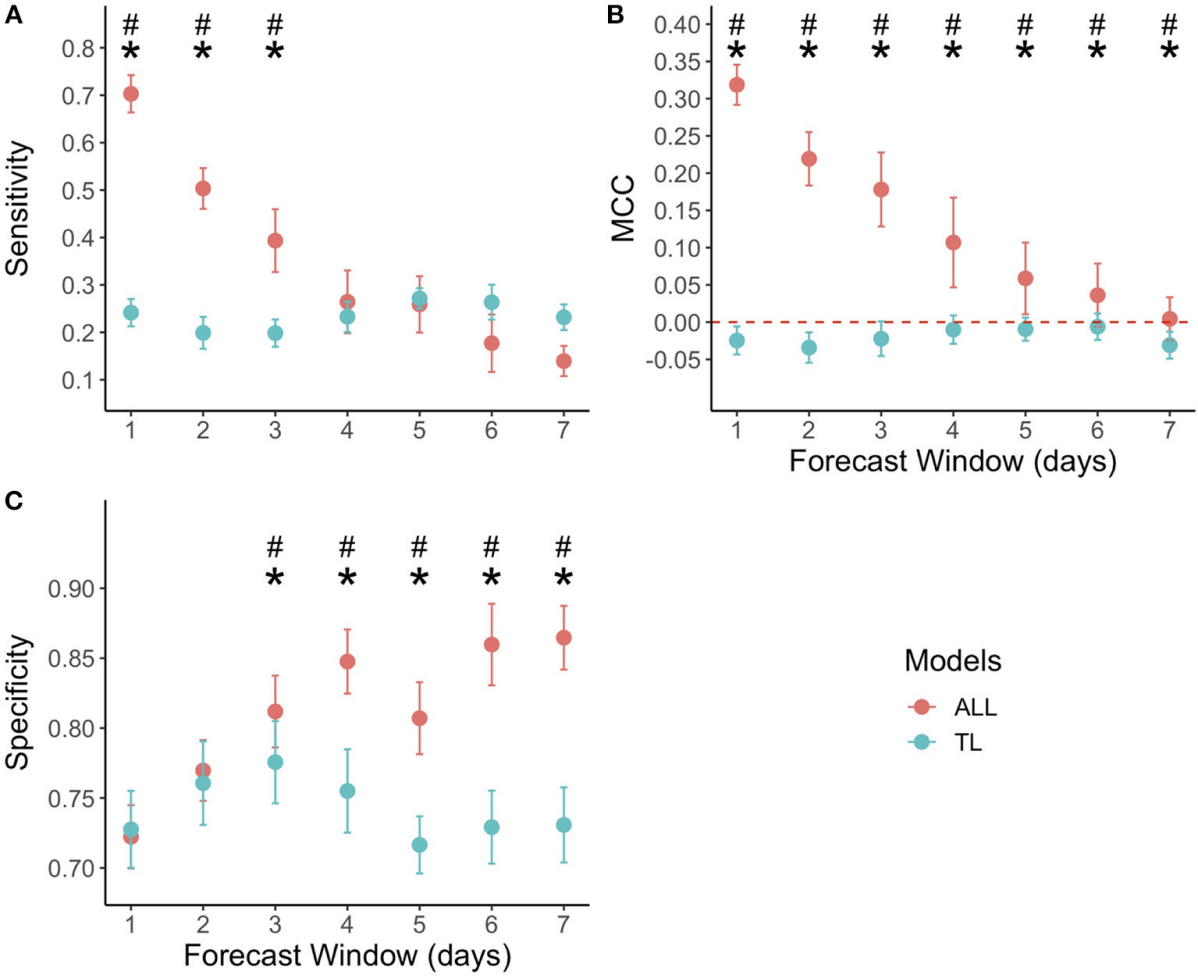
Evaluation metrics scores for both the ALL forecasting models (red dots) and TL forecasting models (blue dots). The x-axis displays the model forecasting window (from 1 to 7 days), while the y-axis presents in **(A)** the sensitivity, **(B)** Matthews Correlation Coefficient (MCC), and **(C)** specificity. The (*) symbol indicates a significant positive difference for the ALL forecasting models compared to the TL forecasting models (*p* < 0.01), as determined by paired *T*-test performed on each individual forecasting window, while the (#) symbol indicates positive large Cohen effect sizes for the ALL forecasting models compared to the TL forecasting models (d > 1). The red dotted line on graph **(B)** indicates a MCC of zero, corresponding to the performance of a model making predictions randomly. Note that the y-axis of each graph are all on different scales to better appreciate the difference between the two types of models for every metric.

Each graph in Figure 3 compares one of the three evaluation metrics derived from the evaluation of the ALL forecasting models and TL forecasting models for each forecasting time window (1–7 days). Two-way ANOVAs performed on sensitivity, MCC and specificity indicated that there was a statistically significant interaction between the models type and the forecasting window (*F*_(6, 406)_ = 351.8, *p* < 0.000 for sensitivity; *F*_(6, 406)_ = 71.38, *p* < 0.000 for specificity; *F*_(6, 406)_ = 186.3, *p* < 0.000 for MCC). Figure 3A compares the two types of model sensitivity, *i.e.*, the number of injury outcomes that the model correctly predicted. On average, the sensitivity was 0.35 ± 0.19 for the ALL forecasting model and 0.23 ± 0.03 for the TL forecasting model. Paired *T*-test performed on each respective forecasting window showed a statistically significant positive effect (*p* < 0.01) on the ALL forecasting models' sensitivity, with large Cohen effect sizes (d > 1) for forecasting windows of 1–3 days. As the forecasting windows got larger, the sensitivity gradually dropped from 0.70 ± 0.03 for a one-day forecasting window to 0.14 ± 0.03 for seven days forecasting window, for the ALL forecasting model. The sensitivity was above 0.5 only for 1 and 2 days forecasting windows. The TL forecasting models' sensitivity remained relatively stable, oscillating between 0.26 and 0.20.

Figure 3B compares the MCC, *i.e*., an index of the models' predictions correlation with real observations. The MCC was on average 0.13 ± 0.11 for the ALL forecasting model and −0.02 ± 0.02 for the TL forecasting model. Paired *T*-test performed on each respective forecasting window showed a statistically significant positive effect (*p* < 0.01) on the ALL forecasting model's MCC, with large Cohen effect sizes (d > 1) for each forecasting window. Every TL forecasting model MCC was below zero, suggesting the model's predictions were slightly worse than if predicted randomly. The ALL forecasting window's MCC decreased as the forecasting windows got larger, reaching 0.00 ± 0.02 for seven-day forecasting windows.

Figure 3C compares the two types of model specificity, *i.e.*, the number of non-injured outcomes that the models predicted correctly. On average, the specificity was 0.81 ± 0.05 for the ALL forecasting model and 0.74 ± 0.03 for the TL forecasting model. Paired *T*-test performed on each forecasting window revealed a significant positive effect (*p* < 0.01) on the ALL forecasting model's specificity with large Cohen effect sizes (d > 1) for forecasting windows of four to seven days. The ALL forecasting model's specificity increased as the forecasting window got larger, reaching a maximum of 0.86 ± 0.02 for seven-day forecasting windows. The TL forecasting model's specificity remained relatively stable, oscillating between 0.76 and 0.81.

**Figure 4:**
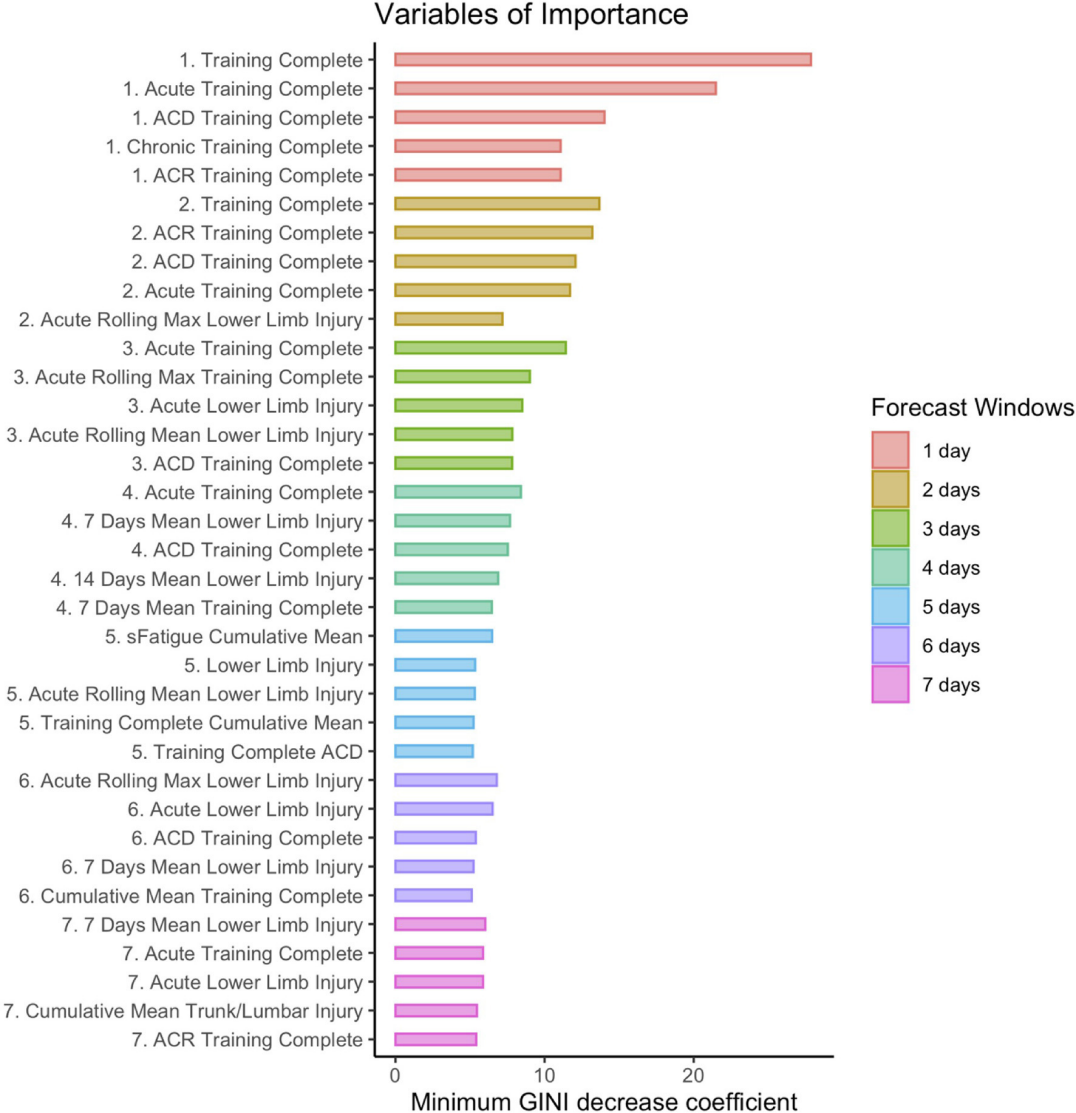
The 5 most important variables for each forecasting window (1 to 7 days) of the ALL forecasting models. The selected variables of importance were the ones with the highest minimum GINI decrease coefficient. The variables are numbered and colored according to their corresponding forecasting time period. ACD, Acute Chronic difference. ACR, Acute to Chronic Ratio.

The ALL forecasting models displayed a significantly higher injury prediction rate and MCC, suggesting they could bring added information to the performance support staff, especially over shorter forecasting time windows of 1 or 2 days, where the MCCs suggest low to moderate agreement between the models' predictions and the observations (MCCs of respectively 0.32 ± 0.03 for a one-day forecast and 0.22 ± 0.04 for a two-day forecast). These models are composed of multiple variables from each of the three components of the injury model (Figure 2). To understand better which categories of variables influence these models, the variables of importance were computed. Figure 4 presents the five most important variables retained from the ALL forecasting models of each forecasting window (1 to 7 days). Every variable in Figure 4 is derived from three monitored variables: athlete training completion, lower limb and trunk/lumbar injury, and sFatigue, an internal training load marker. As the forecasting window gets larger, the minimum GINI decrease coefficient gets smaller.

## Discussion

The results demonstrate that combining a wide array of monitored variables and quantifying the injury etiology conceptual components significantly improve the injury forecasting performance of random forest models. The ALL forecasting models displayed higher MCC than the TL forecasting models. The TL forecasting models' MCC suggests they do not bring any added value to forecast injuries. The ALL forecasting models are particularly promising for injury prediction over short time frames of 1 or 2 days, with sensitivities higher than 0.5 and specificities above 0.7 in both cases. These forecasting models were highly determined by the athletes' training completion, lower limb and trunk/lumbar injury history, as well as sFatigue, a training load marker.

### Machine Learning Framework

The conceptual model of injuries presented in Figure 2 is essential for developing an injury prediction machine learning framework. Practitioners who want to elaborate a data mining system need to have in mind the three conceptual components of injuries, *i.e.*, the stress applied to the athlete, the factors influencing the athletes' stress response, and the stress response itself, which can lead to positive adaptations, or negative outcomes like injuries. They should define key monitoring variables reflecting each component of the conceptual model of injuries, which could vary depending on the context (exercise modality, athletes' level, age, financial resources, etc.) and should be adapted to match the practitioners' needs (Halson, 2014; Starling et al., 2020; Clarsen et al., 2021). Monitored data should be stored in a database, as engineered features based on the data history are essential to reflect the time-dependent nature of injuries (Bittencourt et al., 2016). Handling the database is easier when a problem conceptualization has been made (Figure 2), and a data-mining plan (Figure 1) has been established. This planning provides perspective on the problem and ensures data are monitored and stored purposefully.

Missing data have to be handled (Benson et al., 2021). In this study, we used the simplest imputation form, which replaces the missing data with the most recent measurement. The imputation strategy does not minimize the errors and introduces information loss that could potentially be detrimental to the models' predictive performances. Other imputation strategies that use mean values or regression techniques minimize the models' error prediction (Benson et al., 2021). However, these strategies are less optimal in the perspective of the models' implementation and could introduce higher risks of information leakage and overfitting (Van Eetvelde et al., 2021). To minimize the missing values' imputation detrimental effects on future models' performance, we will be looking at minimizing missing values, by measuring certain variables more frequently. It is a challenge to maximize measurement frequency without overwhelming the athletes (Starling and Lambert, 2018). New monitoring technology to track the athletes' HRV and neuromuscular function may represent an interesting avenue for that purpose (de Zambotti et al., 2019; Roberts et al., 2020). Practitioners are free to use the imputation strategies that better suit their needs. However, they should always keep in mind the models practical value and overfitting risks in their decision-making.

The ALL forecasting models show promising results, especially over short time windows of 1 and 2 days, where more than half the future injury days and more than 70% of future injury-free days can be predicted correctly by the models. Injuries can be compared to extreme meteorological events, which are particularly difficult to forecast (Ghil et al., 2011; Nayak and Ghosh, 2013; Boers et al., 2014). Although modest, the results suggest that, in short time frames, the models could bring added information and help the performance support staff, providing better reaction time and facilitating their injury management decisions. In previous studies, reported sensitivity scores usually ranged between 0.65 and 0.85, while reported specificity scores ranged between 75 and 95% (McCullagh and Whitfort, 2013; Whiteside et al., 2016; Rossi et al., 2017; López-Valenciano et al., 2018; Ayala et al., 2019; Rommers et al., 2020); every study differs in context and methodology.

In this study, methodological choices were made regarding to the models' implementation in the speed skating environment, which could explain the lower sensitivities and specificities. We brought a novel approach that predicts the athletes' health status in upcoming 1 to 7-day time windows. Inevitably, forecasting implies more uncertainty and diminished performance. Injury forecasting was preferred as it has more potential to help and assist the performance support staff. In the experimental setup, we replicated the models' implementation process in the training environment, where models will be trained with all the available information and data history at hand and where new data entries, to predict future outcomes will be provided by the same athletes who participated in the models' training process. Therefore, the test sets data was collected on the same athletes that contributed to train the models. We did not test the models' ability to predict injuries from athletes that didn't contribute to train the models. The training sets' engineered features were computed separately and are not influenced by the test set data. These choices introduce some form of overfitting and information leakage (Van Eetvelde et al., 2021), because new data entries for injury predictions will inevitably be influenced by the athletes' data history used to train the models. However, this process must be seen as a calibration process rather than a major hurdle or methodological issue. The models are calibrated to predict the upcoming injuries of the athletes within the Canadian National Female Short-Track Speed Skating Program and could only work in that particular context, with the specific athletes who took part in the data-mining process. Practitioners should adapt the framework to work in their specific environment and context, and with the athletes participating in their data-mining framework.

### Comparing the Two Types of Models

This study appreciates the significant impact of introducing variables from all three components of the injury conceptual model on the injury forecasting performances. Keeping the methodological aspect constant, the forecasting models, composed of variables from the three components of the injury conceptual model (ALL forecasting models), have displayed significantly higher MCC (*p* < 0.01), with large Cohen effect sizes (d >1) on each of the seven forecasting windows, compared to models built only using training load (TL forecasting models). In fact, the TL forecasting models all had an MCC below zero. Therefore, they behave similarly to a model making predictions randomly and would not be helpful for the performance support staff.

The ALL forecasting models all have an MCC above zero. The MCC values gradually decrease to near zero (0.00 ± 0.02) on 7-day forecasting windows. As the forecasting window gets larger, the ALL forecasting models' ability to predict injury occurrences from the test set decreases. This is expected, especially for complex problems such as injuries, which are influenced by many interrelated variables and some random factors. For instance, injuries can occur accidentally by falling on the ice. These types of injuries are unfortunately impossible to predict. As the forecasting windows get larger, the influence of the different variables and random factors are multiplied, thus explaining the decrease observed in ALL forecasting models' sensitivity and MCC. As for the ALL forecasting models' specificity increase with larger forecasting windows, there are two main reasons. The first one is that patterns leading to an athlete staying healthy are probably better defined and predictable by the models. There are more cases where athletes are healthy, which facilitates pattern recognition. While injuries may be compared to extreme meteorological events, injury-free days resemble day-to-day weather, which is relatively predictable. The second reason is the cost function for injury misclassification, which ties the sensitivity and specificity together. As injuries get harder to predict, the only way to minimize the cost function with larger forecasting windows is to maximize the models' specificity. We applied the same cost function for misclassification on every forecasting window. It would be interesting to test different cost functions depending on the forecasting windows.

### Variables of Importance

It is crucial for the performance support staff to understand the variables that have the strongest influence on athletes' injuries. These variables can provide valuable information on injury risk factors and guide their interventions (Meeuwisse et al., 2007; Clarsen et al., 2021; Van Eetvelde et al., 2021). The random forest algorithms were useful to provide the most important variables [maximizing the minimum GINI decrease (Archer and Kimes, 2008)] of the ALL forecasting models to forecast injuries over the seven time windows. The most important variables are derived from three main features: athletes' ability to complete their training (training completion); lower limb and trunk/lumbar injuries; and sFatigue, an internal training load marker. The training completion variable is intimately tied to our definition of injury (Meeuwisse et al., 2007; Clarsen et al., 2021). Injuries impede the athletes' ability to complete their training because of physical or psychological complaints. Tracking the athletes' ability to complete their training sessions over time is crucial to identify future injuries. Lower limb injuries are another variable worth tracking in short-track speed skating. Lower limb asymmetries have already been pointed out as potential injury risk factors in this sport (Konieczny et al., 2020). In addition, the athletes skate in a crouched posture, which could be responsible for lower back and lumbar discomfort (Hesford et al., 2012). Both variables (training completion, as well as lower limbs and trunk/lumbar injuries) provide indices of the athletes' health status, a factor influencing the athletes' future stress response. The other monitored variables may not be as important for the ALL forecasting models' predictions, but they certainly play a role in the forecasting process. As a matter of fact, sFatigue, an internal training load marker, was among the most important variables for five-day forecasting windows. In addition, as the forecasting windows got larger, the minimum GINI decrease coefficients of the important variables got smaller, potentially linked to the ALL forecasting models' MCC and sensitivity decrease. Practitioners building models in different sports and contexts will necessarily observe different variables of importance.

### Limitations and Future Perspectives

The injury conceptual model presented in Figure 2, although useful to understand the interactions of different categories of variables and their influence, remains simplistic. The etiology of injuries remains much more complex (Meeuwisse et al., 2007; Bittencourt et al., 2016). We will keep improving the conceptual model of injuries, making it more robust and accurate, based on the continuous observations provided by the performance support staff. Improvements to the conceptual model will help orient the data mining framework and future variable monitoring process. Thus far, we have focused on the negative stress aspect of the conceptual model presented in Figure 2 (injuries). However, in the future, basing our work on the same conceptual model, we could explore the positive stress adaptations to predict performance through a similar framework.

The random forest forecasting models act as black boxes. Even if they provide some indicators of the variables of importance, they cannot assist the performance support staff in understanding how each variable influences the injury conceptual model. Nonetheless, the ALL forecasting windows on short time windows (1 or 2 days) are promising, and we believe they could add value to the performance support staff. We plan to implement the models similarly to meteorological models, assisting the performance support staff and providing injury warnings in the upcoming 1 to 7 days. Doing so could facilitate the performance support staff interventions. Much like meteorological models are used in real life, they do not override human judgment. Ultimately, decisions are based on all available information, some of which is not necessarily taken into consideration by the forecasting models. The implementation of the models will be closely documented to help validate and improve our framework.

## Conclusion

In conclusion, the framework presented in this study is a work in progress. It shows promising results for the use of machine learning techniques to forecast sports injury over short forecast time frames of 1 and 2 days. Further work is needed to determine if improved models could help better forecast injuries in time frames of 3 days or longer. The framework is in its early development stages. It will be refined and improved by monitoring additional variables reflecting the conceptual model of injury (Figure 2), gathering more data, and fine-tuning the models' hyperparameters. In addition, the forecasting models were built from the combination of collected data from 11 individual athletes. It would be valuable to individualize the models, as each athlete is different. The variables influencing the conceptual model of injuries, their interaction, and their importance may vary from one athlete to another.

## Practical Applications

- This study presents a novel injury conceptual model (Figure 2), essential to orient variable monitoring and guide data mining projects wishing to predict sports injuries.
- Monitoring different types of variables, quantifying the three components of the conceptual injury model, *i.e.*, the stress applied to the athletes, the factors influencing the stress response, and the stress response itself, improves the injury models' forecasting performances.
- Specifically for athletes within the Canadian National Female Short-Track Speed Skating program who took part in the elaboration of this study's forecasting models, training completion, as well as lower limbs and trunk/lumbar injuries and sFatigue appear particularly important to predict future injuries.
- This study displays promising results from the perspective of forecasting models implementation to assist the Canadian National Female Short-Track Speed Skating program injury prevention process.

## Data Availability Statement

An anonymised version of the raw data (to respect the athletes' privacy) supporting the conclusions of this article will be made available by the authors, without undue reservation

## Ethics Statement

Ethical review and approval was not required for the study on human participants in accordance with the local legislation and institutional requirements. Written informed consent to participate in this study was provided by the participants' legal guardian/next of kin.

## Author Contributions

JB worked on the framework elaboration, model development, conceptualization of the project, and manuscript writing. SD worked on data acquisition, communication with the performance support staff, writing of the manuscript, and conceptualization. SG worked on data acquisition and communication with the performance support staff. FB is the supervisor of the project at the Institut National du Sport du Québec and overviewed each step of the project. All authors contributed to the article and approved the submitted version.

## Funding

This project was funded by the Institut National du Sport du Québec through the *Programme de recherche, d'innovation et de diffusion de l'information* (PRIDI #2).

## Conflict of Interest

The authors declare that the research was conducted in the absence of any commercial or financial relationships that could be construed as a potential conflict of interest.

## Publisher's Note

All claims expressed in this article are solely those of the authors and do not necessarily represent those of their affiliated organizations, or those of the publisher, the editors and the reviewers. Any product that may be evaluated in this article, or claim that may be made by its manufacturer, is not guaranteed or endorsed by the publisher.
